# Liquid Marbles: From Industrial to Medical Applications

**DOI:** 10.3390/molecules23051120

**Published:** 2018-05-09

**Authors:** Roxana-Elena Avrămescu, Mihaela-Violeta Ghica, Cristina Dinu-Pîrvu, Denisa Ioana Udeanu, Lăcrămioara Popa

**Affiliations:** 1 Department of Physical and Colloidal Chemistry, Faculty of Pharmacy, University of Medicine and Pharmacy “Carol Davila”, 020956 Bucharest, Romania; roxana.avramescu@drd.umfcd.ro (R.-E.A.); ecristinaparvu@yahoo.com (C.D.-P.); lacramioara.popa@umfcd.ro (L.P.); 2 Department of Clinical Laboratory and Food Safety, Faculty of Pharmacy, University of Medicine and Pharmacy “Carol Davila”, 020956 Bucharest, Romania; denisaudeanu@gmail.com

**Keywords:** liquid marbles, superficial properties, solid-fluid character, hollow granules, Pickering emulsions, microcapsules, cosmeceuticals

## Abstract

Liquid marbles are versatile structures demonstrating a pseudo-Leidenfrost wetting regime formed by encapsulating microscale volumes of liquid in a particle shell. The liquid core is completely separated from the exterior through air pockets. The external phase consists of hydrophobic particles, in most cases, or hydrophilic ones distributed as aggregates. Their interesting features arise from the double solid-fluid character. Thus, these interesting formations, also known as “dry waters”, have gained attention in surface science. This review paper summarizes a series of proposed formulations, fabrication techniques and properties, in correlation with already discovered and emerging applications. A short general review of the surface properties of powders (contact angle, superficial tension) is proposed, followed by a presentation of liquid marbles’ properties (superficial characteristics, elasticity, self-propulsion etc.). Finally, applications of liquid marbles are discussed, mainly as helpful and yet to be exploited structures in the pharmaceutical and medical field. Innovative pharmaceutical forms (Pickering emulsions) are also means of use taken into account as applications which need further investigation.

## 1. Introduction

Over recent years, surface properties have become very popular among researchers interested in studying special wettable surfaces, like superhydrophobic/superhydrophilic ones. The first part of this review presents a few techniques applied in order to confer roughness to the surface of interest: etching, printing, lithography, etc. Apart from surface structure modifications, coatings are another way to impart special hydrophobic/hydrophilic properties to a solid. Chemical vapor deposition and surface polymerization are some of the methods used to this purpose. A non-wettable structure, that didn’t undergo any surface modifications, formed as a result of interactions between solid particles (external phase) and a liquid (internal phase) is a so called “liquid marble”. Also known as “dry waters”, liquid marbles have attracted scientists’ attention, offering numerous possibilities to exploit their special characteristics in various industrial fields, ranging from the pharmaceutical, medical, chemical, and beauty industries, to micro-/nano-technologies and the newly, still developing environmentally friendly industries concerning water purification.

This review paper includes important aspects in reference to liquid marbles’ composition and applications. New ways to exploit their characteristics in the pharmaceutical domain are proposed. Liquid marbles can be included in a variety of formulations belonging to new generation pharmaceutical forms, like Pickering emulsions. Hypothetically, liquid marbles may be considered as individual pharmaceutical forms, with two components: an active ingredient included into the liquid internal phase and excipients included in the external phase (or the other way around). They can be used for oral or topical administration, depending on their composition and intended therapeutic effect.

## 2. Liquid Marbles

### 2.1. State of the Art

Studying the dynamics of water drops on solid surfaces has become of interest in surface science. Understanding wetting phenomena in correlation with surface affinities for different fluids is crucial. Experimental determination of contact angles formed by a fluid (water is usually used as a model fluid) on a solid surface, offers the possibility to divide surfaces into two categories: hydrophobic and hydrophilic, or superhydrophobic and superhydrophilic, respectively. Analyzing solid surfaces’ wettability through surface structure and roughness, which promote/prevent the sliding of liquid drops, represent the starting point in Mahadevan and Pomeau’s experiments. Experiments were performed following the sliding behavior of mercury droplets on an inclined plane. As a result a new hypothesis was formulated: fluids are easily deforming “entities”, and their movement depends on the interfacial energies and surface roughness. 

In 1999, Mahadevan and Pomeau [1] develop their studies based on the following idea: a viscous fluid droplet can wet a solid support and slide off it when inclined, or it can roll off its surface, without losing its components. For this to happen, the support must be hydrophobic and the liquid needs to have a high surface energy.

**Figure 1:**
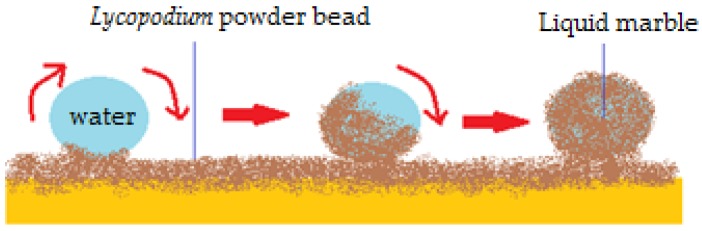
Obtaining liquid marbles by rolling water drops into a *Lycopodium* powder bed.

A similar behavior was signaled by Aussilous and Quéré [2] in 2001 for liquid droplets encapsulated in solid particles, which became known as “liquid marbles”. The first declared liquid marbles were obtained by rolling water droplets (1–10 mm^3^) in a hydrophobic powder bed, made from silica-covered *Lycopodium* microparticles (20 µm). The droplets were immediately covered by particles, maintaining a spherical shape (Figure 1). They were transported onto a glass blade, in order to be compared with plain water drops. Liquid marbles don’t wet the surface since the liquid-solid interface (water-glass) is replaced with a solid-solid interface (*Lycopodium* particles-glass).

Liquid marbles behave as drops placed onto a hydrophobic surface. Thus, they display a high contact angle, minimum friction forces and can easily roll off the support [3]. The double solid-fluid character, explains these denomination of these formations, i.e., “liquid marbles”, “dry water”, “liquid pearls”. Although they achieved the label of “strange objects”, liquid marbles are actually common in some laboratory/industrial procedures, such as granulation of a highly hydrophobic powder. They are also present in everyday life, such as when rain falls on hydrophobic soil after a fire [4], or on Lotus leaves (known as “The Lotus effect”) [5]. In most cases, when artificial liquid marbles are created, hydrophobic powders (polytetrafluorethylene, etc.) are used to cover the drops. Hydrophilic powders (graphene) [6], or even nanofibers made from fluoroalkyl and cellulose acetate co-polymers are also used as external phases, resulting in liquid marbles with superior structural properties compared to those with a hydrophobic powder shell [7]. The liquids used as an internal phase generally display high surface tension, therefore the global surface tension, responsible for the robustness of the liquid marble is also high [3].

### 2.2. Liquid Marbles’ Formulation

An overview shows that liquid marbles have a simple composition: a few microliters of liquid encapsulated in a layer of hydrophobic powder. In order to reveal the necessary conditions to form liquid marbles, this paper takes into discussion powders, as part of the system and also the types of liquids which may constitute the internal phase:

● The external phase

Physically speaking, powders are assemblies of solid particles, with many shapes and sizes, varying between 0.5 and 1000 µm, forming solid-gas disperse systems. Repulsion forces exist between them, due to adsorption at the particle interface. Powders consist of primary particles (individual entities), which can’t be subdivided, and secondary particles, aggregates (formed by joining primary particles at high pressure/temperature) and agglomerates (primary particles kept together by Van der Waals, electrostatic, Newtonian, and non-Newtonian forces).

Powders used to obtain liquid marbles vary in terms of physical properties (particle color, electric charge, wetting degree) and therapeutic activity. A direct correlation between wettability of powders and liquid marbles formation was noticed. The literature shows that hydrophobic powders (both natural: *Lycopodium*, soot [8] and synthetic: polyvinylidene fluoride (PVDF), polytetrafluoro-ethylene (PTFE), polyethylene (PE) [9], polymethylmethacrylate (PMMA) [10], hydrophobic copper powder [11]) and also hydrophilic ones (graphene, carbon black) can be used to cover droplets [3].

The liquid marbles’ wall thickness is given by a combination of mono- and multi-layers of particles. In their experiments, Nguyen et al. [12] show that silica particles larger than 50 µm form monolayers, whilst the smaller ones distribute as multi-layers, forming the liquid marbles’ external phase. After a period of time, the outer layer deteriorates due to reduction of attractive interactions between powder particles. Further morphology analysis reveals an analogy between a liquid marble’s hydrophobic shell, considered to be flexible, and a superhydrophobic surface corresponding to the Cassie-Baxter model. Moreover, the marble’s shell is more complex than the rigid Cassie surface due to its flexibility, which allows stretching and curving to fit the liquid core. In order for the outer layer to stretch when needed, it must have enough powder particles to amend the stretched powder-liquid interface. Fine hydrophobic particles form a multi-layered shell capable of “surviving” any external forces applied on the marble. Thus, fine particles surround the liquid core circumference and help maintain its integrity. A series of modifications occur in the marble’s overall structure as compression forces take action. The fine particles move easily around and pack into newly formed voids, thus maintaining integrity of the shell. The large particles are more rigid, don’t provide flexibility and protection of the internal phase against contact with the support. It was concluded that liquid marbles covered in nanometric particles are more stable, due to their ease of filling any new spaces created during compression. Also, the structure of the liquid marble is given by the Van der Waals forces between the hydrophobic particles, giving it the ability to extend while compressed. There are no hydrogen bonds between the hydrophobic particles, unlike between water molecules, where hydrogen bonds are very strong. Thus, water is allowed to retreat from the inter-particle region, making interactions between shell particles stronger. The resistance of liquid marbles to external deforming forces decreases as the external wall thickens or as the particles are more wettable by water. If the outer layer particles get wetted by the internal phase, then the liquid gains the possibility to infiltrate between spaces, leading to collapse of the marble. These hypotheses are to be noted when designing stable liquid marbles as precursors of hollow granules.

In order to analyze the shell’s structure and layering, experiments on liquid marbles formation were carried out by McEleney et al. [10]. A powder bead made of PMMA and other superhydrophobic copper powders with different particle sizes represented the experimental setup. Liquid marbles formed after water droplets were placed on the clock glass and collected powder particles while tilted. The authors described “a sheet distribution” of particles. Moreover, particles were observed moving across the drop’s surface until they aggregated into a film. When placed onto a PMMA powder bed, marbles formed instantly. The explanation lies in the lower density of PMMA compared to copper powders, translated into smaller forces required to move along the droplet and finally stabilize into a thin film. Although marbles formed on superhydrophobic materials appear to have a monolayered shell, after agitation, particles may distribute into several layers. In case of PMMA, particles form conglomerates and a more a more complex outer shell is formed. A gravimetric analysis reveals a direct proportional variation of powder mass to drop surface area. Large particle sizes result in high powder mass to surface area ratio. This technique is useful in developing a novel method of encapsulating gentamicin sulphate, PMMA bone cement. 

Other studies on liquid marbles outer shell were developed by Bormashenko [13] using optical microscopy, confocal microscopy and SEM techniques. The hypothesis regarding multilayered distribution of powders on the liquid drop surface is confirmed once more, through liquid core clearings separated by *Lycopodium* and PVDF particles, which were observed on experimental liquid marbles.

Moving on to a small-scale analysis of liquid marbles’ external phases, it is stated that particles with the same hydrophobicity will exhibit attractive forces between them due to the relative signs of curvature of the liquid-vapor interface menisci at the particle surface. This implies that particles inducing menisci with alike curvatures attract and those inducing menisci with opposite curvatures repel. It is assumed that this phenomenon drives particles into forming aggregates at the surface of water drops, instead of maintaining separation [14].

Analysis of liquid marbles’ shells behavior was carried out in high temperature conditions, in order to observe structural modifications. The evaporation process of the internal phase will be analyzed also in Section 4.4, regarding structural modifications of the whole liquid marble. Evaporation of the internal phase takes place, inducing transition stages of the outer layer. During liquid marbles’ high temperature exposure, its shell undergoes certain changes, in terms of rigidity, as a consequence of particle agglomeration. These modifications are staged as follows: immediately after formation—“liquid stage”—described as a free structure with low superficial tension; the “light bonded stage” which follows as evaporation begins and the whole structure becomes rigid; the “hard solid stage”, a transition from the organized to the unorganized stage, preceding the “wrinkled stage” and collapse [15,16].

● Liquid marbles’ special architecture

Liquid marbles’ architecture can vary depending on the properties of the shell and internal phase. When analyzing special liquid marbles’ formulations, their components should be noted in terms of superficial properties, rugossity, geometrical arrangement, homogeneity, when referring to the shell and viscosity, surface energy, polarity, behavior in electric/magnetic field, when concerning the internal phase. Many types of innovative coatings with various applications were developed, as well as compartamented marbles formed by slightly compressing drops together. Some of these recently discovered liquid marbles are presented next, along with experimental observations. 

An example of liquid marbles with a particular shell includes as internal phase a copper sulfate solution and external phase-hydrophobic poly-high internal phase emulsion-polymer (HIPE) (containing a mixture of HDBMA_76_: EGDMA_24_, hexafluorbutylmethacrylate: ethylene glycol dimethylmethacrylate). Thereby, the shell is a porous structure, with particles interconnected by gigapores of micron dimensions. They resemble natural organisms like radiolarians (protozoa producing mineral microtubes) or diatoms (microalgae with cells interconnected by tubes), as shown by Gokmen et al. [17]. After water evaporates and the HIPE-shell is removed, a copper sulfate sphere is left behind. This method may represent a model in designing spherical objects, whereas the interconnected channel system functions as a distributor of external solutions 

Among unconventional liquid marble coatings, those made from carbon nanotubes and fullerenes (C_60_) are also innovative. These coatings cover an internal phase containing reactants. When liquid marbles float at the surface of a liquid and are exposed to IR radiations, they release the reactants, as a result of light being transformed into heat. The reactants interact with substances from the liquid, resulting in new chemical products [18].

Shells that increase liquid marbles’ resistance to compression were developed from fluorocarboxylic copolymers and cellulose hydrophobic nanofibers. Other coatings (Fe_3_O_4_/C micro-sheets) decrease the evaporation process of the internal phase and successfully function as microreactors in nanocomposite synthesis [19].

**Figure 2:**
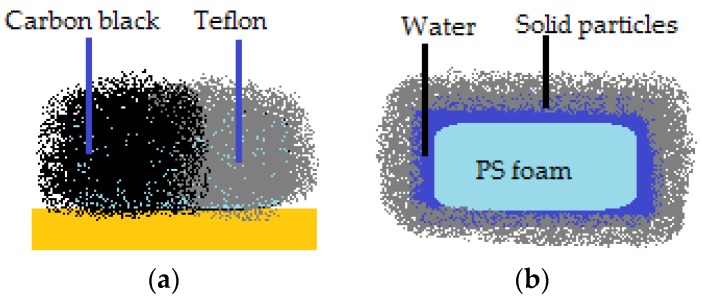
(**a**) Liquid marble resembling a Janus particle; (**b**) Cubic FPS marble.

The possibility to create a liquid marble covered in a shell that doesn’t have the same properties on the whole surface, also exists. They resemble Janus particles and can be guided by electric fields. Half of the coating (spherical cap) is made from carbon black (semi-conductor), whilst the other half is made of Teflon (dielectric), as displayed in Figure 2a [20]. The Janus marble is formed after two different marbles (i.e., one covered in carbon balck and the other in Teflon) are forced together in order for coalescence to happen. Progresses was registered in the field of composite liquid marbles with special shapes, containing organic and inorganic fluids, as shown by Bormashenko et al. [21]. Thus, cubic-shaped droplets were obtained, as illustrated in Figure 2b. 

**Figure 3:**
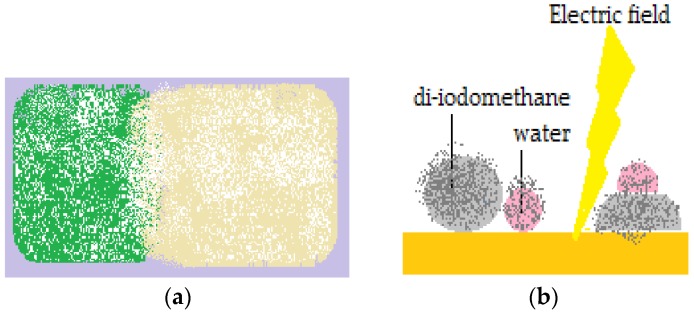
(**a**) Cubic *Lycopodium* marbles placed in contact; (**b**) Water marble climbing on to a diiodomethane one, sharing the same PTFE shell.

Cubic foamed-polystyrene (FPS) particles were plasma-treated in order to increase their surface energy and hydrophilicity, wetted with water and coated with hydrophobic colloidal particles. Shaped liquid marbles maintained stability both on a solid and liquid supports. These special shaped marbles do not coalesce upon contact, due to the fact that hydrophilic particle coated liquids behave as elastic solids, and not fluids. Uncommon geometrical figures are formed after two marbles are compressed together, as displayed in Figure 3a. The non-stick drop composite category is completed by water and di-iodomethane droplets covered in PTFE, which are slightly compressed together. The resulted formation shares the hydrophobic shell. As an electric field is applied, the water droplet climbs onto the di-iodomethane one which is denser, and the structure is re-shaped, as presented in Figure 3b [22].

Special techniques, such as chemical vapor deposition (CVD), are applied to ionic core liquid marbles, in order to obtain a complete polymeric shell. Although the technique is usually applied to solid surfaces in order to cover them in thin films, Bradley et al. [23] used it to cover droplets. The thermodynamical stability of the system is given by surface tension and solubility effects. The condition for achieving stable polymeric-coated marbles relates to the surface energy, which should allow spreading of the polymer over the entire core liquid. It was demonstrated that in order for polymerization to happen at the liquid core’s surface, the monomer should not be soluble in the liquid. Otherwise, since the monomer establishes the polymerization site, the reaction would occur both on the liquid-air interface and inside the core liquid, which is an unwanted process. The CVD technique promises emerging applications in optics, sensing and developing hybrid materials. Experiments carried out by the same authors, revealed that the polymeric shell’s height and roughness are inversely proportional to the liquid core’s viscosity and polymer deposition rate. By varying these parameters, CVD can be used to obtain microstructured film shells is suitable for potential applications in tissue engineering, electrolyte membranes and separations [24]. Some changes in the shells’ architecture result in increasing liquid marbles’ stability. Examples show transforming from a granular arrangement into a thin film by exposing to solvent vapors, or peptide units self-assembling triggered by pH changes in the interior of the drop’s shell. Besides polymeric hollow capsules, fatty acid and triacylglycerol crystal based liquid marbles are transformed into capsules through heating [14]. 

● The internal phase

Liquid marbles typically include high surface tension liquids such as water or glycerol. Xue et al. and Matsukuma et al. report the possibility to obtain liquid marbles using low surface tension liquids (dimethyl sulfoxide–DMSO, toluene, hexadecane, ethanol [25], methanol, 1,4-dioxane [26]) by adapting the powder properties chosen to form the coating. The possibility to “build” non-adhesive drops from a hydrophobic powder covering ethanol, diiodomethane, is given by the liquid’s concentration and its behavior in contact with the powder (the powder is either engulfed by the liquid or it remains at the gas-liquid interface, forming a shell) [9].

In microfluidics, ionic liquids such as sulfuric acid aqueous solutions, sodium hydroxide, diiodomethane, hexadecane, blood, are encapsulated by oligomeric tetrafluoroethylene (OTFE), forming liquid marbles stable to the action of a mechanical force [27]. Organic solvents can also be encapsulated by fluorinated decyl polyhedral oligomeric sesquisiloxane (FD-POSS) [25].

A particular case of liquid marbles is that of Galinstan (an eutectic liquid mixture mainly consisting of gallium, indium, tin) covered in Teflon, isolators (SiO_2_) or semiconductors (CuO, ZnO, WO_3_). They have the ability to roll, to float on water and can be used as semiconducting systems, resistant to high temperatures and to impacts. Their incomplete oxide shell prevents particle rearrangement, making them different from conventional liquid marbles, when obtained in air. It is preferable to obtain them in a diluted hydrochloric acid solution, as a reduction reaction takes place [28].

● Other components

In order for the shell to be stable, liquid marbles’ formulation includes auxiliary substances such as binders. Obtaining hollow granules from liquid marbles by evaporating the internal phase requires using such binders, which maintain the integrity of the outer shell. Among binder solutions we recall polyvinylpyrrolidone (PVP), hydroxypropylmethyl cellulose (HPMC), and hydroxypropyl cellulose (HPC), with concentrations varying between 2% and 18%, polyethylene glycol (PEG) 200, PEG 300, PEG 400, PEG 600, etc. [29,30].

### 2.3. Liquid Marbles Covered in a Hydrophilic Shell

It is well known that hydrophilic particles are covered by water, with no possibility to switch places in order to obtain liquid marbles, although a metastable state can occur, when hydrophilic particles aggregate and include air in the newly formed structures. The droplet is held captive and maintains its shape. A big amount of energy is required in order to destroy the barrier formed by particle aggregates and air, in a regime similar to superhydrophobic surfaces corresponding to the Cassie-Baxter model [3].

### 2.4. How to Obtain Liquid Marbles

The most popular method used to obtain liquid marbles is the one proposed by Aussilous and Quéré: rolling a liquid droplet on a hydrophobic powder bed, so that the particles form a shell on its surface; the overall aspect is similar to a marmorated pearl. This method has its limitations, being time consuming, with irregular coverage, unreproducible and the impossibility to obtain drops smaller than 10 µL, which are more stable. These are some of the reasons why liquid marbles are not yet produced at an industrial scale. Methods are still in the research phase, mainly in terms of reproducibility and efficiency [3]. Generally, the principal aim is to uniformly cover the drop with particles. Techniques that only cover a part of the drop with micronic particles (trimethylsilyl chloride) were reported. The droplet is gently placed on the powder bed, without rotating it. The trend of the droplet is to shrink and acquire a spherical shape. Subsequent evaporation determines some particle’s to float around the drop, covering it [31]. If the particles are big (PMMA, hydrophobic copper powder ~ 320 µm) they can’t encapsulate the whole drop. Liquid marbles have a different appearance in this case, displaying an open area on the upper part [10]. 

In 2012, a new method, based on condensation and drop nucleation was reported. The liquid representing the internal phase (water, glycerol, ethylene glycol) is placed in a warm-up container with a heat source placed underneath. A thin layer of hydrophobic particles (Cab-O-Sil fumed silica and micronic sized Teflon) is described at the liquid-air interface. As the liquid boils, the vapors condense and get covered by hydrophobic particles. Micronic sized pearls of liquid are formed, representing micronic liquid marbles. By separately studying these liquid marbles, another phenomenon is displayed: by heating “parent-marbles”, much smaller liquid droplets called “child-liquid marbles”, are formed at their surface. The phenomenon is referred to as “liquid marble sweating”. The “child-marbles” roll off the “parent-marbles” and are more robust (3–1000 µm), depending on the heating temperature. This method has some advantages, as it affords very small (3 µm) liquid marbles and the possibility to control temperature and the duration of exposure to heat, depending on the desired result. It is an industrially applicable technique [32]. 

A promising technique to obtain liquid marbles with different applications (controlled drug release, water purification membranes) involves wrapping drops in transparent glass fibers, which prevent fluid evaporation [33].

Another automatized and efficient process to produce liquid marbles is based on the vibration of a container and gathering of covered droplets immediately after formation. The method allows parameter adjustment and the use of more than one hydrophobic powder [34].

A revolutionary method which doesn’t use hydrophobic particles, involves coverage of the drop after an impact in a cloth of superhydrophobic nanofibers. Resulting liquid marbles exhibit resistance to evaporation, to vortex mixing in an oil phase, with the advantage of no internal phase loss [7].

Liquid marbles with diverse formulations can be obtained through various methods. That’s why choosing the appropriate components and experimental parameters is a very challenging process. In order to test liquid marbles properties in correlation with different formulations, comparative experiments were carried out. Liquid marbles with two designs were involved: a type covered with fumed silica nanoparticles and another with Teflon nanoparticles. The results indicate that they both are transparent, more robust and resistant to compression forces compared to liquid marbles covered in microparticles. These properties arise from long intercalate particle chains formed at the drop’s surface [31].

Among recently proposed materials to cover liquid marbles are: graphene (Fe_3_O_4_/C) micro-sheets covering non-volatile liquids, with successful applications as reaction catalysts [19]. Also, polymeric latexes (PS, PMMA) stabilized with polyionic liquids proved to form a floating resistant shell [35].

## 3. Powders’ Superficial Properties

### Contact Angle: Methods of Determination

Powders included in liquid marble formulations, mostly hydrophobic, should be characterized mainly by establishing their wettability. The first experimental determination involves calculating the contact angle described at the liquid–powder interface. This leads to another important parameter, which is the surface tension. These determinations are important in anticipating a liquid marble’s resistance over time or under certain conditions and also help establish new formulations.

It is well known that the contact angle is described by a drop of liquid, at the liquid-solid-air interface. A contact angle at equilibrium, $\theta_{e}$ > 90° corresponds to a hydrophobic surface, respectively <90° to a hydrophilic one and can be calculated using Young’s equation (Equation (1)). The contact angle depends on the surface tensions delimiting the system. Surface tension determines the drop’s shape, as a result of uncompensated forces of the molecules at the edge of the drop. It practically turns the drop into a sphere:

(1) $$cos\theta_{e}=\left(\gamma_{SA}-\gamma_{SL}\right)/\gamma_{LA}$$

From a wettability point of view, a powder is best characterized by the contact angle, although there are some unwanted inconveniences because of porosity. The contact angle can be experimentally determined using many methods. The most popular is the “sessile drop” method. It’s simple, reproducible and uses a small amount of powder. A drop is formed with a syringe. It falls on the powder bed and the contact angle is measured by a goniometer. 

Another technique, less applied in case of powders, is the Wilhelmy method, used to calculate small contact angles (<20°). It requires compacting a powder on a plate surface, so that it adheres without the use of any adhesives. The plate goes vertically into a liquid tank. The measurement consists of the force needed in order to pull out/insert the plate. That force has to overcome the weight of the plate, but also the superficial tension of the liquid. Some inconveniences lead to inexact measurements: the liquid is absorbed by pores and the compacted powder endures denaturation [36,37].

The Washburn technique, also known as the “liquid penetration and capillary rise method” is also based on powder compaction. It measures the penetration rate of a liquid through a powder compacted into a capillary tube. The method is applicable only theoretically for qualitative determinations. Theories suggest that the drop penetrates the powder bed vertically, reaching a depth at witch capillary pressure balances the liquid weight on the column [36,37].

There is a hypothesis suggesting that by not taking into account the pore radius between the particles, these theories are all wrong. The contact angle is a unique characteristic of every single system, capable of imposing its own wetting regime, so that every situation corresponds to one of the above wettability measuring techniques. All the presented methods have advantages and disadvantages and can be adjusted depending on the powder [24].

When characterizing a powder from a wettability point of view, besides measuring contact angles, superficial tension is also an important parameter. Among the methods used to calculate superficial tension, the following can be mentioned: drop height measurement, shape analysis, and the vibration method.

## 4. Liquid Marbles’ Properties

### 4.1. Surface Tension

Given the fact that liquid marbles are structures completely separated from the support through a particle shell, they demonstrate a pseudo-Leidenfrost wetting regime. Thus, the Young equation does not apply in their case. Authors have proposed a series of means to determine liquid marbles’ surface tension by including a “hypothetical” contact angle, assuming an imaginary liquid-solid contact line exists between the liquid marble and the support [38].

Aussillous and Quéré [2] and Bormashenko et al. [39] state that measuring the drop’s height is a simple way to determine surface tension of liquid marbles, in correlation with the fact that at small volumes they are spherical, and as the volume increases, they flatten. Surface tension $\gamma_{e_{ff}},\;$ can be calculated using the following equation (Equation (2)):

(2) $$\gamma_{eff}=\frac{\rho gH^{2}}{4}$$

where ρ is the liquid density and H  represents the liquid marble’s maximum height.

The drop’s shape analysis is made only through empirical ways, by calculations, because the drop’s shape is influenced by gravity. Thus, a drop is considered to be a flattened spheroid, rather than a perfectly shaped sphere. Geometrical parameters are measured after fitting the drop into an ellipse shape [40].

Measurements of drops heights are correlated with shape analysis, in order to determine surface tensions. Big liquid marbles are used because they flatten due to gravity and capillary forces. Liquid marble’s height variation in time depending on its radius is shown by Newton et al. [41] through a graphical representation. This interpretation helps select the exact volume needed to obtain a perfectly spherical marble, corresponding to a maximum contact angle (>180°).

Determining a liquid marble’s surface tension through the vibration method was suggested by Bormashenko et al. [42] and Celestini and Kofman [43]. The unique feature of this method is that a liquid marble is placed on a vertically vibrating platform. The resonance frequency corresponding to a maximum deformity of the marble is measured. Surface tension $\gamma_{eff}\;$ is determined using the following equation:

(3) $$\gamma_{eff}=\frac{2\pi \rho Vf^{2}}{h\left(\theta \right)\left(1-cos\theta \right)}$$

where f stands for resonance frequency, *V* is the drop volume, θ  is the actual value of a “hypothetical” contact angle, and h(θ)  is a multiplication factor.

All the previously mentioned methods to determine liquid marbles’ surface tension, rely on establishing this value for the liquid drop, not considering that the particles in the shell influence the determination. Thus, the surface tension varies due to interactions between particles covering the drop. Experimental values obtained using these three methods vary for the same substance as follows: for a liquid marble covered in Teflon, height measuring method revealed $\gamma_{eff}$ ~ 60 mJ/m^2^, shape analysis $\gamma_{eff}$ ~ 53 mJ/m^2^, and vibration method $\gamma_{eff}$ ~ 43 mJ/m^2^ [3].

The notion of surface tension attributed to a complex formation such as a liquid marble, remains unclear. Experimental studies show that it would be erroneous to assign a certain surface tension value to a liquid marble. That is because liquid marbles go through intermediate changes (from the moment of formation until collapse) and also because particles display a one/multi-layered distribution, an aleatory/welded setting while forming the outer shell.

Some approaches regarding liquid marble’s surface tension take into account the particles forming the shell. Considering a layer of perfectly spherical and smooth particles with $R_{p}$ radius; they are projected towards the exterior of the drop (into the air) at a distance  d, given by Equation (4):

(4) $$d=R_{p}\left(1-cos\;\theta \right)$$

where θ stands for the actual value of the “hypothetical” contact angle described at the particle-drop-air interface [8,44].

For the particle to get attached to the drop, a part of the liquid-air interface area $A_{LA}\;$ and a part of the solid-air interface area $\;A_{SA}$, are being replaced with the solid-liquid interface area $A_{SL}=A_{SA}$. Once attached to the drop, the particle’s energy, Δf, changes. It can be determined using the following Equation (5):

(5) $$\Delta f=A_{SA}\left(\gamma_{SL}-\gamma_{SA}\right)-A_{LA}\gamma_{LA}$$

Considering the equilibrium contact angle given by Young’s equation (Equation (1)), the surface free energy of the particle becomes Equation (6):

(6) $$\Delta f=-A_{SA}\gamma_{LA}\left(cos\;\theta_{e}+A_{LA}/A_{SA}\right)$$

The ratio between liquid-air/solid-air interfaces will have a positive value, as follows (Equation (7)):

(7) $$A_{LA}/A_{SA}=\left(1-cos\;\theta_{e}\right)/2$$

The energy of the particle always decreases when it adheres to a drop surface, in favor of liquid marble formation [45,46]. The actual value of the “hypothetical” contact angle and also surface energy will change if the particle is forced into/off the drop. The surface free energy becomes Equation (8):

(8) $$\Delta f=\pi R_{p}{}^{2}\gamma_{LA}{\left(cos\;\theta_{e}-cos\;\theta \right)}^{2}$$

Proposed models considering drops covered in a one-layered smooth and spherical particle shell don’t take into account interactions between particles and theorize the idea according to which liquid marbles covered in hydrophobic particles ($\theta_{e}$ > 90°) are more stable [4].

Other approaches by Arbatan et al. [38], on determining liquid marble’s surface tension, regard liquid marbles as reservoirs, protected by solid shells. The whole spherical formation is characterized by a Laplace tension. In order to measure this tension, a capillary tube of radius *r*, vertically introduced in a water tank is considered. The capillary rise (h)  is calculated as follows (Equation (9)):

(9) $$\frac{2\gamma cos\;\theta }{r}-\rho gh=0$$

In the same situation, with the difference that the capillary tube is introduced into a liquid drop of radius *r*, an increase in capillary height is observed (presuming that the radius *r* remains constant while the drop is forced into the tube). The height difference (Δ*h*) between this and the previous case is described by the following Equation (10):

(10) $$\frac{2\gamma }{R_{e}}+\frac{2\gamma cos\;\theta }{r}-\rho g(h+\Delta h)=0$$

where γ—liquid’s surface tension, *θ*—actual value of a “hypothetical” contact angle described at the liquid-tube-air interface, ρ—liquid’s density, *g*—gravitational acceleration, $R_{e}$—liquid drop radius, h—capillary rise.

If the drop radius is bigger than the capillary rise, then gravity is responsible for the drop’s shape, and the equation (Equation (11)) will take into account $r_{1}$ and $r_{2\;}$ as curvature radiuses. Thus:

(11) $$\gamma (\frac{1}{r_{1}}+\frac{1}{r_{2}})+\frac{2\gamma cos\;\theta }{r}-\rho g(h+\Delta h)=0$$

### 4.2. Liquid Marbles’ Elasticity

Experimental liquid marbles formed by rolling a liquid drop on a powder bed, display different properties from those formed naturally, such as when raindrops fall on a hydrophobic soil. Drops fallen from great hights are covered by particles during shape distortion, due to internal currents and to kinetic energy resulting from the fall [47]. The resulting liquid marbles have properties similar to elastic solids and also to fluids. The first step in anticipating a liquid marble’s behavior consisted in analyzing solid particles, of almost identical diameters, placed on a liquid surface. Vella et al. [48] completed the experiment which became a two-dimensional granular model. *Lycopodium* and graphite particles spread at the liquid’s surface, forming a mono-layered shell, with properties similar to an elastic solid, depending on the particle’s agglomeration geometry.

**Figure 4:**
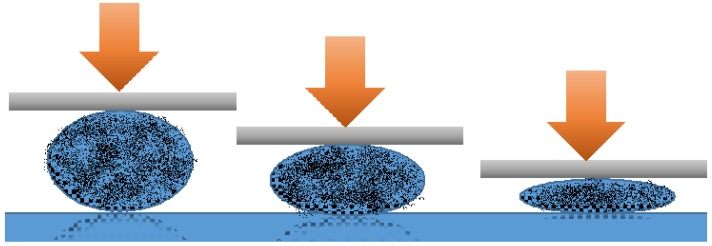
Liquid marble’s progressive deformation determined by increasing compression forces.

In order to test liquid marbles’ double character hypothesis, laboratory-made liquid marbles were compressed. Formulations included ultra-high molecular weight polyethylene (UHMWPE) as shell-forming particles and potassium chloride 100 mM as core liquid. The liquid marbles obtained by Samuel et al. [49] were gradually compressed, as shown in Figure 4. Video recordings showed the modifications suffered by liquid marbles, proportional with the compression force applied. Results indicated the following changes: when a small compression force was applied, liquid marbles were slightly deformed; as the force increased, the shell began to crack, until the maximum force determined the drop’s collapse. The experiment demonstrated that liquid marbles’ elasticity gained through the liquid meniscus formed between the shell’s particles, allows a compression up to 30% from the drop’s initial dimension.

**Figure 5:**
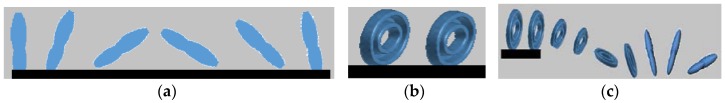
Liquid marbles rolling off an inclined plane. (**a**) “Peanut” shape; (**b**) “Doughnut” shape; (**c**) Transformation of “doughnut” into “peanut” shape, as the plane is removed.

It is assumed that liquid marbles’ elasticity is related to the lack of contact between the liquid core and the solid support, due to the particle coating. The absence of colored traces left by sodium hydroxide liquid marbles rolled on a phenolphthalein surface, proves this hypothesis [50]. Analysis of speed variation function droplet ratio, for liquid marbles with a viscous core placed on an inclined plane (4° and 24°) reveals the following: in the most inclined plane case, speed is higher and deformations are more obvious in bigger liquid marbles. Video images by Aussilous and Quéré [2,51,52,53] show that centrifugal forces are responsible for the shape of rolling liquid marbles. As the droplet rolls off a less inclined plane, its shape resembles a peanut (Figure 5a). As the plane becomes more inclined, the drop accelerates and acquires a toroidal (“doughnut”) shape (Figure 5b). The center is not completely empty as the liquid is not fully eccentrically distributed. The doughnut shape is a metastable one, evolving into a peanut shape, as the inclined plane is removed (Figure 5c). Similar forms were reported in diamagnetically levitating drops. Studies on liquid marble’s deformability continue, thus they exhibit many applications in zero gravity tests [53].

### 4.3. Liquid Marbles’ Coalescence

**Figure 6:**
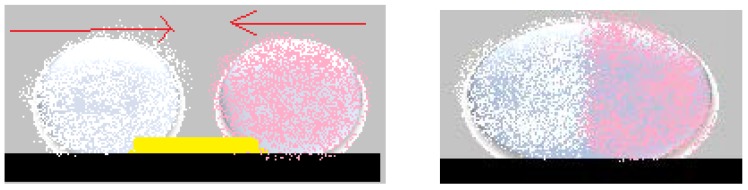
Coalescence of liquid marbles covered in *Lycopodium*(pink), respectively in PVDF (white), through a glass bar.

A strong link can be established between liquid marble’s elasticity, presented above, and coalescence of the droplets. Coalescing liquid marbles result in complex formations, capable of incorporating extraneous objects. Coalescence does not happen by its self, but rather by applying exterior compression forces. To illustrate these aspects, experiments were carried out using the following materials: as liquid marble coatings polyvinylidene fluoride (PVDF), polytetrafluoro-ethylene (PTFE), polyethylene (PE), *Lycopodium*, SiO_2_, fluorinated decyl polyhedral oligomeric sesquisiloxane (FD-POSS), and carbon black. The internal phase included distilled water colored with potassium permanganate, making it easier to notice the coalescence. A hydrophobized (through radiofrequency plasma procedure) cylindrical glass bar is used to connect the two liquid marbles and to favor the coalescence. The result is a single non-adhesive drop, with the bar included inside, as shown by Bormashenko et al. [54] (Figure 6).

**Figure 7:**
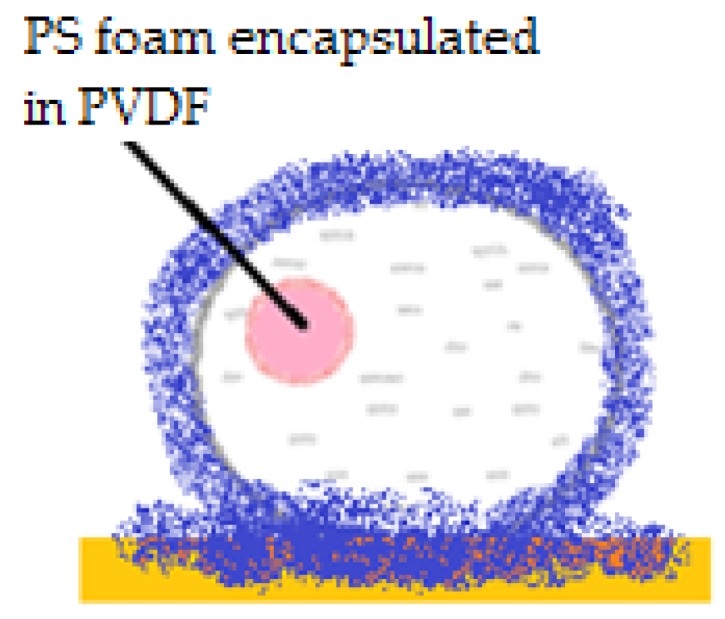
“Sandwich” liquid marble including PS foam covered in PVDF.

**Figure 8:**
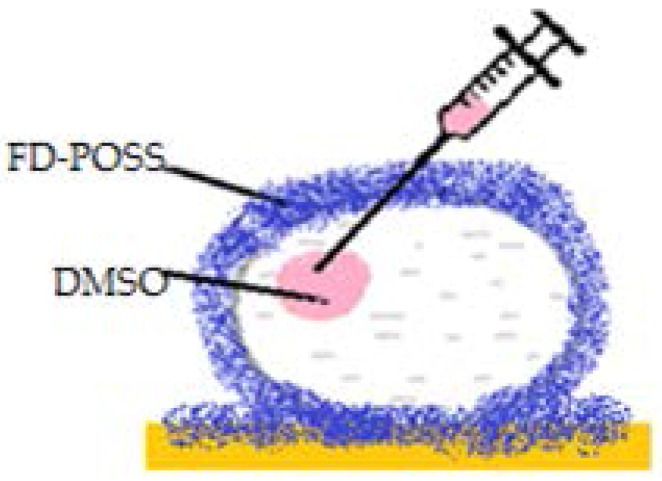
Liquid marble made from hexadecane/toluene, covered by FD-POSS, encapsulating DMSO.

An interesting aspect worth mentioning about liquid marbles that swallow extraneous objects, is the fact that they can include millimetric particles of hydrophobized polystyrene (PS) foam. The PS is wetted with water, and the resulted drops are covered with PVDF hydrophobic particles. “Sandwich liquid marbles” are obtained, as presented by Bormashenko et al. [54]. (Figure 7). The particularity of these liquid marbles is that they can “engulf” solids, as well as liquids, respecting the condition of immiscibility. Two experimental steps are followed: forming liquid marbles with an organic fluid core, covered by FD-POSS; injecting the previously obtained marble with another organic fluid. Different pairs of organic liquids are proposed: toluene/DMSO, hexadecane/water, hexadecane/DMSO, etc. (Figure 8). 

Coalescence of liquid marbles connected through a glass bar represents a track to obtaining liquid marbles resembling Janus particles i.e., marbles with shells delimited into two zones with different hydrophilicities: one hemisphere is dielectrical and the other is a semiconductor (carbon black) (Figure 3). The entire formation is activated by an electric field [20]. “Sandwich liquid marbles” including solid particles and also organic liquids have many applications in the biomedical field (liquid marbles with separate compartments, containing microorganisms, cells, reactants) [55,56,57].

### 4.4. Liquid Marbles’ Drying

Compared to plain liquid droplets, liquid marbles have a core fluid protected by a porous shell, which delays the internal phase’s evaporation. It is well known that once evaporation starts, the drop’s volume decreases and the solid film thickens, due to particle compression. Particle welding was also observed in Leidenfrost droplets [58]. Changes during evaporation of the internal phase were also discussed in Section 2.2, while presenting “the external phase” of liquid marbles, from a structural shell modification point of view.

**Figure 9:**
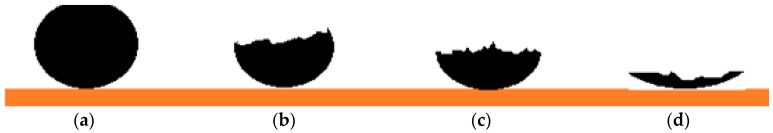
Plan and horizontal views of PTFE liquid marbles during evaporation: (**a**) initial liquid marble; (**b**) the marble begins to shrink as the shell wrinkles; (**c**) the outer shell is thikend and bulked; (**d**) liquid marble collapse.

Tosun et al. [59] measured and compared PTFE marbles’ evaporation rates with that of pure water droplets. The observations indicated how PTFE aggregates are formed at the liquid-air interface, finally displaying as a 3D multi-layered network. The PTFE coating shows some small empty areas where the core is exposed. Hydrophobic aggregates in the shell make it harder for water to evaporate. At some point, the aggregates begin to approach one another and water starts to evaporate. Consequently, the drop’s volume decreases and a shrinkage process is triggered along with the shell’s thickening. As the evaporation process continues, liquid marbles become undefined shapes with a wrinkled appearance. A few stages of PTFE liquid marbles’ evaporation are presented in Figure 9. The results indicate that PTFE microparticles function successfully as a liquid marble protective shell, especially when water is evaporated slowly. Emerging applications in microfluidics, genetic analysis etc. are proposed for PTFE marbles which proved a longer lifetime before any architecture changes occur.

Bhosale et al. [31] developed a comparative study on water evaporation from micronized Teflon (µPTFE), fumed silica nanoparticles and dimethyldichlorosilane (nDMDCS)-covered droplets. Fumed silica forms long chains at the surface of the droplet, resulting in networks which confer mechanical resistance, transparency and a delayed evaporation rates.

The evaporation rate of liquid marbles covered in graphite was also investigated. The study concerned the time elapsed until the shells’ particles welded. A comparison was made between liquid marbles with different internal phases: 0.9 mM sodium dodecyl sulfate (SDS) surfactant and SDS: water mixtures. The time was larger in case of the SDS: water mixture. SDS improved liquid marbles’ stability through evaporation, but only when used in a small concentration (0.9 mM). Otherwise, if the concentration was bigger, hydrophilicity prevented formation of and decreased the stability of liquid marbles [60]. The same authors developed experiments using 11 solutions of different SDS concentrations and a Teflon-FEP hydrophobic substrate. They demonstrated a difference between evaporation rates, as follows: for concentrations up to 80 mM SDS-evaporation takes place without modification of the contact angle, while for concentrations >200 mM SDS-evaporation followed a constant regime of solid-liquid contact, due to droplets being rapidly fixed to the substrate [61].

Liquid marble evaporation studies required mandatory measurements of an initial contact angle and of the contact angle after evaporation or in the presence of a heated stand. Liquids with a small contact angle (~10°) don’t undergo a proper evaporation process, because of the strong interactions with the substrate. Likewise, as a drop is placed on a heated support, the margins cool, and evaporation can’t be exactly evaluated [62]. The lifetime of a drop decreases with the increase of the support’s temperature. By the time the stand reaches ~200 °C (Leidenfrost transition temperature), the drop’s lifetime increases, due to the isolating vapor substrate at the liquid-vapor interface [14]. Experiments demonstrate these statements using liquid marbles covered in graphite (10–20 µm), heated at 100 °C–465 °C [63]. Different results were expected when referring to aggregate/non-aggregate particles or isolator/non-isolator particles. Surprisingly, the isolating particle layer transforms liquid marbles into superhydrophobic structures. Also, a superhydrophobic heated stand induces the drop’s base evaporation, resulting in a vapor layer (like the Leidenfrost effect). This layer collapses on an ordinary support, but not on a superhydrophobic one, which also increases the drop’s stability at temperatures higher than the boiling point [64]. 

Monitoring the ambient temperature and humidity should be considered while measuring evaporation rates. The more humid the air, the more delayed is the evaporation of liquid marble’s internal phase. Also, the superhydrophobic stand’s geometry and the chemical structure of the particles covering the drop, are important parameters influencing the evaporation rate [65]. Liquid marble’s shell and its layers also raise questions. A monolayered shell made from spherical micron-sized particles determines liquid marbles to dry faster than an uncovered drop. The solid particles don’t allow interface compression during drying, compared to the uncovered drop situation, where heat generates shrinkage at the liquid-air interface. Multi-layered liquid marbles dry harder than uncovered drops, depending on the particle layer’s thickness [62].

### 4.5. Liquid Marbles’ Freezing

**Figure 10:**
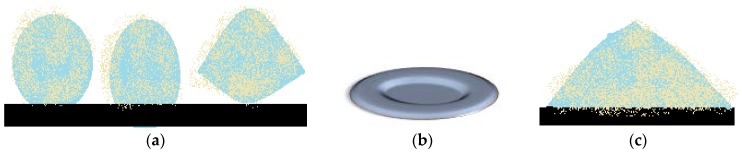
Shape changes in freezing liquid marbles. (**a**) initial shape, “dome” and “flying saucer” shape; (**b**) central “crater”; (**c**) “pointy peak”.

As previously presented, experiments regarding liquid marble’s behavior in extreme temperatures, show that they react similarly to Leidenfrost drops, due to the outer shell which prevents heat transfer between the support and the liquid core. When referring to low temperatures, the first reported liquid marbles subjected to frost were reported in 2012. The formulation included deionized water covered with *Lycopodium* powder. They were placed on a silicone support and cooled at −8 °C. Throughout the cooling process, liquid marbles undergo shape changes, height reduction and outward extension of the sides. As shown by Hashmi et al. [66]. Deformation processes include the “dome” shape, which evolves into a “flying saucer” shape (Figure 10a). Freezing starts at the bottom of the drop and extends to the top. The same experiment was made using an uncovered water drop, placed on a superhydrophobic support. As the freezing process advances, the drop extends its base and develops a protrusion at the top. In the same experimental conditions, a *Lycopodium* covered drop, forms a central crater (Figure 10b). An uncovered drop in the same conditions as the one covered in *Lycopodium*, manifests an opposite behavior, developing a pointy peak (Figure 10c).

The study of freezing liquid marble’s dynamics, lead to the following conclusions: unlike the uncovered drop, a liquid marble behaved differently, at the same temperature; the “flying saucer” shape emerged as a result of drop flattening and outward extension of the edges; shape changes appear due to the freeze inhibiting properties of the hydrophobic coating and to the Marangoni effect-internal phase convection flow along the internal margins of the drop [66].

Drops covered in hydrophobic particles have similar shapes to drops placed on superhydrophobic stands. Liquid marbles covered in less hydrophobic particles, develop a lateral projection. When cooling droplets covered in hydrophilic particles, water has the tendency to form ice aggregates in the cavities between the particles. Drops covered in hydrophobic particles, display surface protrusions (aggregates), as earlier described [67].

### 4.6. Liquid Marbles’ Floating and Self-Propulsion

Liquid marbles at room temperature or subjected to high temperatures are known to undergo certain changes in their architecture, as presented in Section 4.2. Evaporation of the liquid core takes place along with thickening and wrinkling of the external phase, resulting in imminent collapse. This behavior is studied while marbles are placed on a solid support. More recent studies were carried out considering liquid marbles’ behavior while floating on a liquid surface. It is well known how a solid particle induces liquid surface’s deformation, due to gravity and surface tension. The case of floating liquid marbles required a lot more analysis, because not only the liquid support surface gets deformed, but also the marble itself.

**Figure 11:**
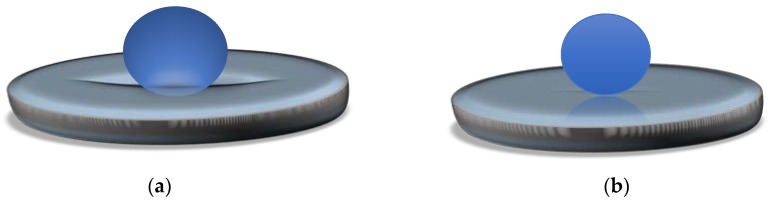
(**a**) Distilled water marble floating on water surface; (**b**) Water marble floating on NaCl aqueous solution.

Experiments began by considering a difference between two effective surface tensions: the one describing the air-marble interface and the one characterizing the liquid support-marble interface. Differences appear due to the fact that particles covering the marble change their distribution while packed between two fluids–the liquid marble’s core and the carrier. Results show how floating marbles collapse while floating on a liquid surface, and consequently lose their core, by releasing it into the liquid carrier. This phenomenon required more investigations. Preliminary studies reveal that compared to a liquid marble placed in air, one maintained in a humid environment maintained its floatability many days [68]. Also, in terms of distilled water marbles floating on water surface, it was found that as drop volume increases, the deformation of the water-air interface becomes more pronounced, as illustrated in Figure 11a. For smaller distilled water marbles floating on NaCl water solutions this deformation becomes negligible, as shown in Figure 11b [69].

**Figure 12:**
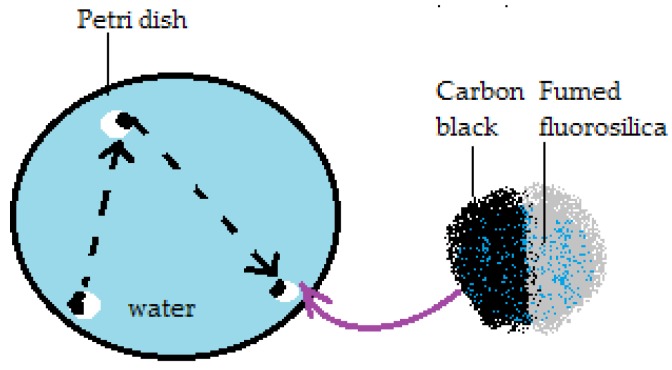
Self-propelled Janus liquid marbles’ movement.

Extended research on floating liquid marbles arrived in the point where besides floating, possible marbles’ movement on a liquid surface was taken into consideration. Thus self-propelling liquid marbles have gained scientists’ attention. By the self-propelling phenomenon, an autonomous movement is implied and correlated by authors [70] with the Leidenfrost effect and the Marangoni solutocapillary flow. The experimental setup for liquid marble self-propulsion studies included: water and alcohol aqueous solution as core liquids, extremely hydrophobic fumed silica as shells and Petri dishes with water, glycerol, silicone oil and aqueous ethanol solutions as liquid supports. Results indicate that marbles placed onto silicone oil collapsed immediately, whilst those placed on glycerol floated. Rapid and straight-line movement was observed for marbles on water surfaces. The reason why glycerol and water maintained liquid marbles’ integrity lies in their different viscosities yet close surface tensions. It was noted that the internal core of the liquid marble didn’t touch the liquid supporting surface. Proof comes form the fact that when phenolphthalein marbles self-propelled on a water-NaOH solution, they did not leave any colored traces. Thus, liquid marbles are supported by a vapor layer, similar to the one observed in the Leidenfrost effect’s case. This vapor layer starts to form as ethanol from the marbles’ core begins to evaporate, much faster than water. The liquid marble loses contact with the supporting liquid, as a gaseous film (ethanol vapors) separates them and enables further evaporation. Liquid marbles become unstable and are guided along the surface by a Marangoni flow resulting as alcohol condenses on the water’s surface. The tendency of liquid marbles to move faster with the increase of alcohol concentration in the liquid core was experimentally demonstrated. Thus, their movement will be suppressed if alcohol is added to the supporting liquid. It was also demonstrated that marbles did not roll while moving. For this purpose, Janus-marbles covered in fumed silica powder and carbon black were used. Straight line movements were observed with no traces left by the carbon black hemisphere, as presented in Figure 12. Liquid marbles’ self-propelled motion is stopped due to water drag, which proved to be larger than air friction. Among suggested future applications of self-propelled liquid marbles targeted drug delivery systems, lab-on-chip devices and microsurgery attract researchers’ interest, especially in the medical and pharmaceutical field. 

## 5. Liquid Marbles’ Applications

Liquid marbles exhibit promising applications in numerous domains like the pharmaceutical and medical industry, biochemistry, microfluidics, microreactions, chemistry and biotechnology. Next, this essay will summarize some of liquid marbles’ already known applications and will propose some new pathways in order to exploit their special properties.

### 5.1. Applications in the Pharmaceutical Domain

The pharmaceutical industry is dealing with a problem regarding hydrophobic powder granulation. The standard procedure implies granule formation as a result of liquid distribution around solid particles, when aggregates are formed. If the powder is hydrophobic, then it covers the liquid, resulting in a liquid marble. This physico-chemical incompatibility can be transformed into an advantage. Liquid marble-obtaining processes can undergo adjustments so that the resulting formations can be used to design particle assemblies, with many possible pharmaceutical applications: primary packaging, or as integrated parts of formulations [71,72].

● Liquid marbles as hollow granule precursors

Emptied and dried liquid marbles are considered hollow granule precursors. The first step in this direction was made by Khanmohammandi et al. using as model powders salicylic acid, *para*-ethoxybenzamide, and glass Ballotini spheres. Water, glycerol, PEG 200, 300, 400, 600 were included in liquid marble formulations as internal phases and binders, respectively. Experiments show that many conditions need to be fulfilled to obtain stable, spherical liquid marbles, which may function as hollow granule precursors. The fluid drops need to be bigger than the powder particles and should maintain their shape after impact with the powder bed. The fluid must form with the support a contact angle higher than 90°. It is mandatory for the powder to cover the drop’s surface, forming a layer thick enough to isolate the fluid from the exterior. The shell is formed through currents generated inside the drop after impact, or by particle migration due to the kinetic energy of the fall. If the drop is rolled in a powder bed or is transported onto another support, it needs to be robust enough to survive this process without losing components or changing shape. If these conditions are fulfilled, the liquid marbles thus obtained are basic in obtaining pharmaceutical products with hydrophobic active ingredients. This approach offers the possibility to adapt and control working parameters (particle dimension and structure, liquid flow), depending on the desired final product [29,30].

**Figure 13:**
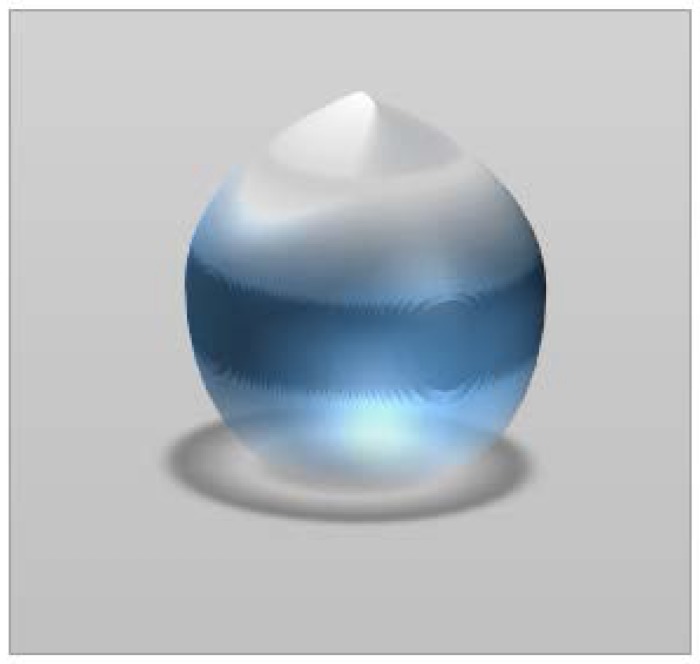
Hollow granule obtained from aerosil and HPMC, dried at 100 °C.

Hollow granules obtained through solid spread nucleation can be compressed in order to obtain tablets. Experiments use powders such as: polytetrafluoroethylene (PTFE), aerosil; other components include Ballotini spheres and binders (PVP, HPMC, HPC). Liquid marbles with this formulation are dried through different methods (in moist air at 24 °C, freezing at −50 °C, in dry air at 60 °C, 80 °C, 100 °C). Results show that a high drying temperature, nanometric particles and high binder concentrations promote hollow granule formation. Liquid marbles’ survival and quality are being evaluated through these experiments. HPMC proves to be the best binder in high concentration, forming liquid marbles which survive various temperatures, independent of the shell. HPC is compatible with Ballotini spheres, used as shells, while PVP are the same with PTFE. Aerosil is successful regarding spherical shape generation, when used together with HPMC and drying at 100 °C, resulting in hollow granules with an ideal shape, as presented by Khanmohammadi et al. [30] (Figure 13). As expected, liquid marbles shrink if dried at high temperatures, reaching the same dimension as the initial drop. Another important aspect is that low temperature drying is beneficial when using low binder concentrations.

Considering all these observations, liquid marbles are gaining great importance in the pharmaceutical industry as precursors of hollow granules, tablets, emulsions. They can incorporate low solubility and hydrophobic active ingredients and represent alternatives in the case of substance incompatibilities or targeted release drugs (ex: intestine and not stomach). The active ingredient’s protection is mandatory against local acidity/enzymes, pathogens/other substances competing for binding sites and can be achieved by choosing the ideal development process, while following Quality by Design Guidelines.

● Liquid marbles as microcapsule precursors

**Figure 14:**
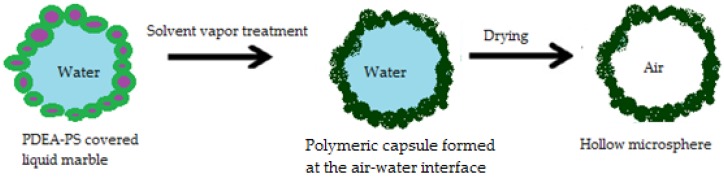
Transformation of a liquid marble into a microcapsule.

Other pharmaceutical forms related to liquid marbles are microcapsules. Water droplets rolled in a submicrometer size polystyrene particles (PDEA-PS) powder bed (carrying poly 2-(diethyl- amino)ethyl methacrylate) and dried at high temperatures, lose their internal phase through evaporation, as previously presented. The process resembles a balloon’s deflation. Even so, experiments show that once exposed to solvent vapors, these liquid marbles transform into polymeric capsules, due to PDEA-PS’s plasticization effect. These capsules maintain their shape even after complete internal phase evaporation. The complete transformation process resumed by Ueno et al. [73] is presented in Figure 14. Capsules containing organic/inorganic compounds or colloidal crystals are obtained from liquid marbles with SiO_2_ dispersions. They represent important candidates for obtaining capsules with modified/delayed/targeted drug release 

● Liquid marbles as part of Pickering emulsion formulations

After Pickering first reported them in 1907, emulsions with the same didn’t receive attention for a long period of time [74]. In 2004, Pickering emulsions regained importance, as water drops covered in silica nanoparticles, also called “dry water” or “water in air emulsions”. In the food industry, many scientists work with lipid crystals adsorbed to the surface of drops immersed in a liquid. Those are in fact, Pickering emulsions. 

**Figure 15:**
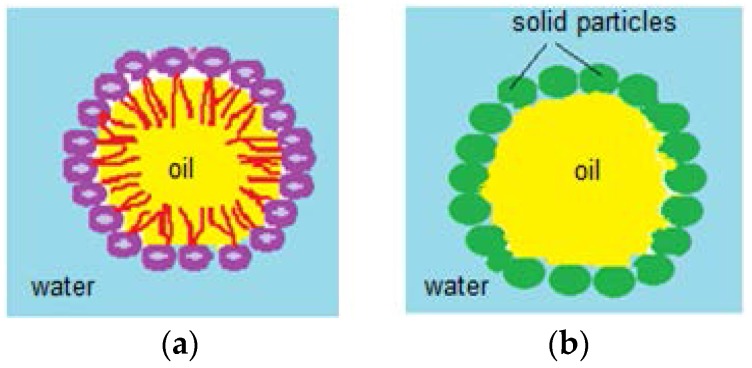
(**a**) Oil/water emulsion; (**b**) Pickering oil/water emulsion.

Pickering emulsions are represented by water in oil and oil in water emulsions, as shown in Figure 15. They can also be multiphase emulsions, where the surfactant, used as stabilizer, is replaced with surface adsorbed solid particles. Among Pickering emulsions’ advantages, applications in pharmaceutical and cosmetic fields are important, due to coalescence resistance and lack of irritating surfactants. By adsorbing nanometric (~100 nm)/micronic solid particles at the drop’s surface, these emulsions gain stability. Although the adsorbed particles are not amphiphilic, they are strongly attached to the drop they cover, due to partial water and oil wetting which confers integrity of the immersed droplet (in any kind of aqueous/oily medium) [75,76]. Stabilizer particles like calcium carbonate, clays, latexes, magnetic particles, carbon nanotubes, carbon black, copolymeric micelles, pH sensitive particles, proteins, spores and even bacteria are also components of Pickering emulsions. These stabilizers undergo surface modification processes, such as chemical treatments, in order to gain hydrophobicity and to confer stability to the drops. Pickering emulsions’ stability is influenced by the hydrophobicity of the solid particles adsorbed at the water/oil interface and by the oil’s chemical properties. Hydrophobic particles stabilize water/oil emulsions, whilst hydrophilic ones stabilize oil/water emulsions [75].

**Figure 16:**
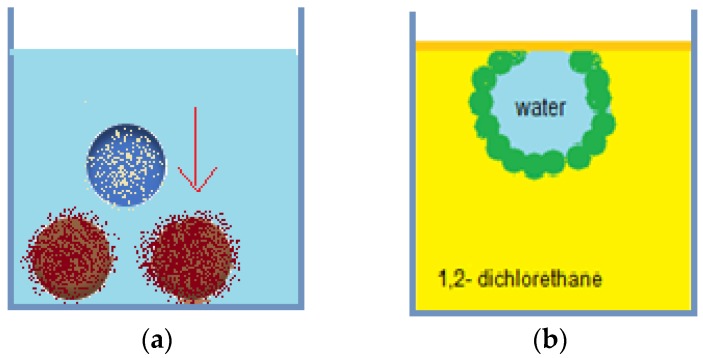
(**a**) *Lycopodium* covered liquid marbles submerged in PDMS; (**b**) Liquid marbles anchored at air/1,2-dichlorethane interface.

Liquid marbles are an integral part of Pickering emulsions, and their versatility allows their inclusion in complex systems, depending on their formulation and properties. Experiments regarding Pickering emulsions’ stability and other properties use as raw materials the following: water, glycerol-internal phase, PTFE, PVDF, PE, carbon black, Lycopodium-external phase. After obtaining liquid marbles by rolling the drops in powder beads, their behavior is studied after immersion, and at the surface of toluene, xylene mixture, carbon tetrachloride, dichloromethane, 1,2-dichloroethane, chloroform, DMSO, DMF, acetone, ethanol, PDMS. Results by Bormashenko et al. [77] indicated good liquid marbles stability in less polar liquids (PDMS, toluene, xylene mixture, chlorinated solvents) as presented in Figure 16a and total collapse in polar liquids (DMF, DMSO, ethanol, acetone).

By placing liquid marbles on a liquid’s surface, the following observations were made: submerging in silicone fluids and aromatic solvents resulted in maintaining stability at the bottom of the tank; submerging in chlorinated solvents resulted in anchoring at the solvent/air interface Figure 16b; liquid marble’s destruction at contact with polar solvents. Water/oil Pickering emulsions include as an external phase: silicone fluids, aromatic solvents, chlorinated solvents. Polar solvents (DMF, DMSO, ethanol, acetone) don’t allow Pickering emulsion formation. Pickering emulsions find their applicability as encapsulating devices for active ingredients, without the necessity to include possibly irritating, cytotoxic or hemolytic surfactants in the formulation. Models proposed as special coatings (allowing targeted transport and release) include: particles covered in hydroxyapatite/polymers, obtaining bio-degradable microspheres [78], hybrid lipid-silica microcapsules [79]. Regarding topical use of Pickering emulsions with caffeine, studies reveal that the hydrophobic molecule’s absorption is faster compared to other pharmaceutical forms. The explanation stands in liquid marbles covered in a silica shell, promoter of caffeine absorption from the aqueous phase of the emulsion into the epidermis [80].

Studies using all-*trans* retinol as a hydrophobic active ingredient model, follow its absorption rate from Pickering emulsions in comparison with a water/oil emulsion stabilized with surfactants. All-*trans*-retinol accumulated in the corneous layer (lipidic medium) of the epidermis in both cases. A higher percentage corresponded to the Pickering emulsion, which did not penetrate the lower layers of the epidermis, due to the silica particles included in the formulation. The same type of investigation showed that retinol included in oily particles of Pickering emulsions, is stabilized against UV radiation. Biodegradable Pickering emulsions have emerging applications in the food industry and represent environmentally friendly drug delivery systems [76].

● Liquid marbles resembling Janus particles

Janus particles are divided into two different hemispheres. The differences between them refer to wettability, optical, electrical, magnetic properties. Depending on anisotropic properties, they work as emulsion stabilizers, surfactant analogues, optical probes for chemical, biological, rheological measurements [81]. Janus particle’s ability to reduce interfacial tension is due to their amphiphilic character. In 2006, gold and iron oxide covered Janus particles were compared, by measuring the capacity to reduce interfacial tension of *n*-hexane and water. They proved to have an increased surface activity compared to their corresponding particles, confirming the ability to stabilize Pickering emulsions [82]. Liquid marbles work as microreactors in dopamine polymerization at the air/water interface. Asymmetrical deposition of a polydopamine film leads to formation of drops resembling Janus particles. Dopamine’s functional groups lead to newly discovered applications of PD/SiO_2_ covered particles in chelation and reduction of metallic ions [83].

### 5.2. Liquid Marbles as Cosmeceuticals

The term “liquid marble” is often replaced by “dry water” or “magic powder” within the marketing strategies used by the cosmetic industry. These denominations come from the special abilities of the products. After topical use, the initial feel is a powder-like touch, followed by moisture and hydration given by the fluid component. Liquid marbles draw attention in the beauty products industries thanks to their easy manipulation, capacity to incorporate big amounts of liquid in small volumes and keeping the interior fluid intact. In most cases, liquid marbles are included in foundation formulas and hair products. In 2015, Yue et al. [84] start a study regarding the validity term of these products and their stability, linked to their high water percentage. Mass market products were tested. Results show that foundations contain less water and a high amount of coloring agents; majority of the tested products contain film promoters (co-polyvidone VP/VA), which prolong liquid marbles’ life; a smaller life time than the one stipulated on the package was observed for products containing liquid marbles. The evaporation rate of the internal phase is decisive in establishing liquid marble’s and also cosmetic products life time. Studies will be continued in this direction.

Liquid marble formulations represent a basis for obtaining foundation, make-up products, antiperspirants/deodorants, solar protection products, and last but not least, drugs. Among the advantages of these products, the most important are easy topical application, followed by a moisture and cooling sensation due to internal phase liberation. Formulations include deionized water or floral water (50–90%) mixed with polymers/co-polymers (PVP, methacrylate), wetting agents (hyaluronic acid), hydrosoluble vitamins, antiperspirants, deodorants, preservatives, antioxidants. Natural or organic dyes can be added. The liquid components are mixed together separately from the solid ones. Afterwards both phases are brought together to form the final product. These cosmetic products are suitable for oily skin, due to a low content in oily phase (under 1%). Therapeutic agents which can be included in the formula are: antifungal, antibacterial, antiviral, anesthetics, analgesic, keratolytic, antidandruff, antidermatitic, antipruritic, antiseborrheic, antihistaminic, pigmentation/depigmentation agents, vitamins, corticosteroids, and hormone substances [85].

Considering liquid marbles’ previously discussed shell versatility and physical properties (elasticity, permeability), undoubtable explanations arise on their capacity to function as miniature chemical, biochemical, biological reactors, with numerous applications in domains relying on reactions (chemical, biological, etc.).

### 5.3. Liquid Marbles as Biological and Biochemical Micro-Reactors

**Figure 17:**
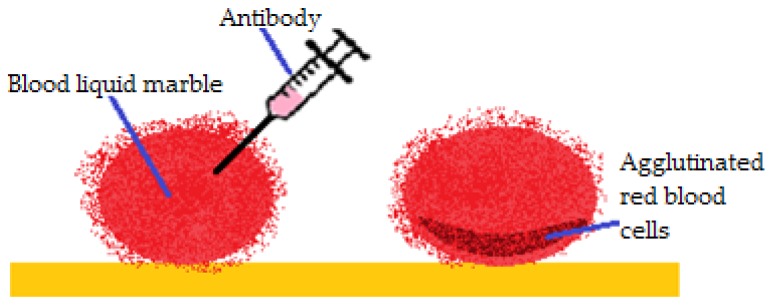
Haemagglutination reaction inside a blood liquid marble.

Arbatan et al. [55] studied liquid marbles as micro-bioreactors used to determine blood types. Human blood drops were covered in hydrophobic precipitated calcium carbonate treated with stearic acid. Antibody solutions (anti-A, anti-B, anti-D) were injected within the drops. The change of color (from red to dark red) was monitored, as an indicator of the haemagglutination reaction. The results were immediate and visible. In a few seconds the color changed at the bottom of the blood drop, as presented in Figure 17. If color change didn’t occur, the test was considered negative for the tested antibody and another blood type could be tested. Advantages of the method include: low cost, little volumes of reagents and samples; the possibility to control the reaction by drop coalescence or reactant injection and low contamination risk because everything happens within the marble.

**Figure 18:**
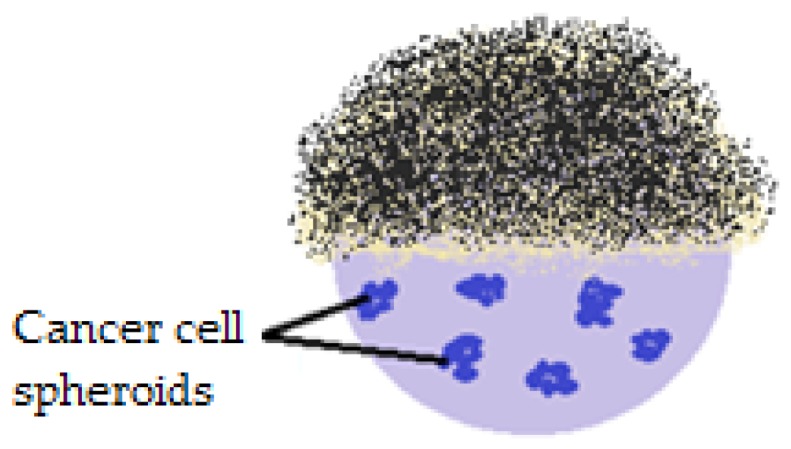
PTFE covered liquid marbles (section) used to observe cancer cell interactions in time.

The first study demonstrating that liquid marbles can be used to obtain in vitro spherical/spheroid cancer cell aggregates was developed in 2012. The experiments involved using Teflon covered liquid marbles, inoculated with HepG2 hepatocellular cancer cells (Figure 18). 

The main idea was that the cells form aggregates within liquid marbles, reflecting human tumor physiology better than 2D cell cultures. Ten days after HepG2 cell inoculation, cells aggregation was successful, due to the restricted environment and to the integrity of the PTFE shell (small surface tension and reduced adhesive tendency). Human intervention was minimum, as liquid marbles were incubated through the experiments. In order to analyze the cells inside the drops, covering them with magnetic particles was proposed. A magnetic force applied at the bottom of the drop, releases the top and makes an analysis of the internal phase possible. Nutrients and waste exchange are permissible and are developed using a micropipette. This method gained interest in tissue engineering, in obtaining embryoid bodies, observing and understanding cell interaction mechanisms [86].

A new simple and reproductive method which eliminates classical inconveniences of obtaining embryoid bodies consists of using liquid marbles as bioreactors. Stem cells can evolve into embryoid bodies, in vitro, under well-established conditions, with the possibility to differentiate into endothelial, cardiac, neuronal, hematopoietic tissues. Liquid marbles offer the necessary conditions for embryonic cells to evolve into embryoid bodies. The transformation’s efficiency is higher as the inoculum is richer in cells and liquid marbles are bigger [56].

Formation of embryoid bodies represents a model technique used to induce in vitro sheep oocyte maturation. Liquid marbles are covered in PTFE and contain oocyte complexes. The small internal phase offers ideal contact between cells and stimulates cell aggregation as well as their maturation. This technique offers the possibility to remove metabolites during embryo maturation, to morphologically evaluate them and select vial oocytes for cryogenic programs [87].

Following this direction, more recent reports refer to liquid marbles as “biotechnological devices” used to conserve mammalian cells through cryopreservation. The method uses Teflon- covered liquid marbles containing murine fibroblasts, reference cells used in cytotoxicity studies. The study establishes election parameters to be followed in order to preserve, recover and also maintain mammalian cells morphology, viability, adhesion and proliferation capacity [88].

As earlier presented, liquid marbles possess attributes which recommend them as miniature culture media: internal phase volume adjustment (a few to hundreds of µL), liquids mix only if they are forced to coalesce, and not if they are kept apart; the shell’s permeability for gases allows aerobic microorganism culture. These properties were tested on human and animal cells, and also on microorganisms: *Lactococcus lactis* spp. *cremonis* (Gram positive bacteria, aerotolerant anaerobic, used widely in dairy industry) and *Saccharomyces cerevisiae* (brewer’s yeast, an optionally aerobic, model microorganism used to study microbiological processes). Teflon covered liquid marbles (20 µL) were inoculated with strains of these microorganisms and kept at rest (30 °C) for 24 h. Liquid marbles were analyzed immediately after formation and also after 24 h. In order to minimize evaporation rate, liquid marbles were kept in Petri dishes, surrounded by water droplets. Results show shrinkage of liquid marbles during incubation, indicating a small loss of water. The shape maintained during the rest of the period. Both *L. cremonis* and *S. cerevisiae* liquid marbles show an increase in number of individuals, due to shell’s permeability for oxygen and carbon dioxide. The advantage of using liquid marbles as culture media is the favorable air/volume ratio, which allows rapid proliferation of aerobic organisms, compared to a classical culture media, in the same conditions. As expected, liquid marbles are culture environments with low proliferation rates for aerobic microorganisms. Liquid marbles which can maintain life in nitrogen, hydrogen disulfide media are still developing [57].

### 5.4. Liquid Marbles as Chemical Reactors

Special structural properties of liquid marbles, such as internal 3D space with adjustable volume, separated from the external environment through a non-adhesive membrane, permeability for oxygen and carbon dioxide, transforms them into successful biological micro-reactors. When it comes to chemical reactions occurring separated from the environment, liquid marbles are presented as chemical reactors.

Miao et al. [89] proved the ability of liquid marbles covered in silver nanofibers (hydrophobized with perfluorodecanthiol) to function as chemical reactors. The internal phase includes methylene blue and sodium borohydride (NaBH_4_). They undergo a degradation process (catalytic reduction) with a good yield, due to the silver shell, which has two roles: provides catalytic sites and a bridge for electron’s transfer between NaBH_4_-nucleophile and methylene blue-electrophile. The reaction is monitored by determining the concentration of reactants. The shell is reusable, maintaining its properties. The method proves to be economic, efficient and applicable in the case of toxic reactants.

**Figure 19:**
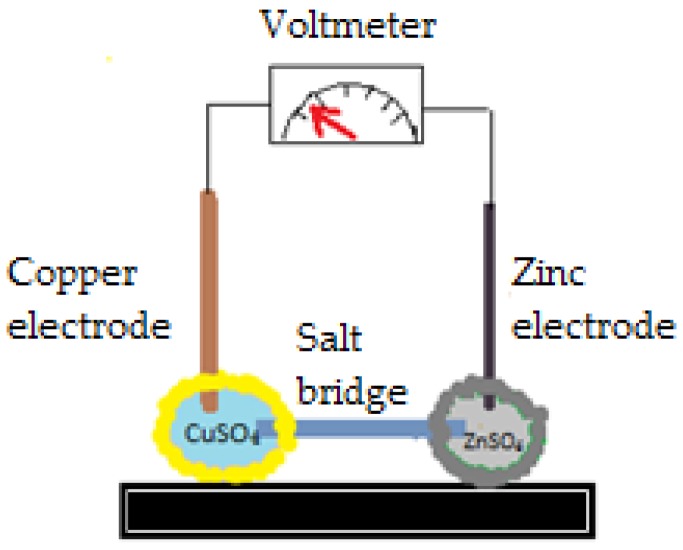
Liquid marbles included in a Danielle cell.

Including liquid marbles in a Danielle cell is a method proposed by Li et al. [90], which also allows initiation of chemical reactions. Two liquid marbles take the place of the galvanic cells, linked by a salt bridge (facilitates electric charge transport) and by the corresponding electrodes (Zn^2+^ and Cu^2+^). Electrolyte solutions include ZnSO_4_, CuSO_4_, as presented in Figure 19. Three liquid marbles pairs were connected in series. A charge exchange occurs and an electrical current capable of lighting a light bulb (potential > 3 V) was generated. Electrolyte concentrations were varied and electrolyte solution intake and uptake pumps were attached. This model represents a preliminary study in understanding mass transfer and mixing conditions of the samples. It’s a first step in adjusting necessary experimental conditions required to use liquid marbles as a Danielle cell and as chemical microreactors.

**Figure 20:**
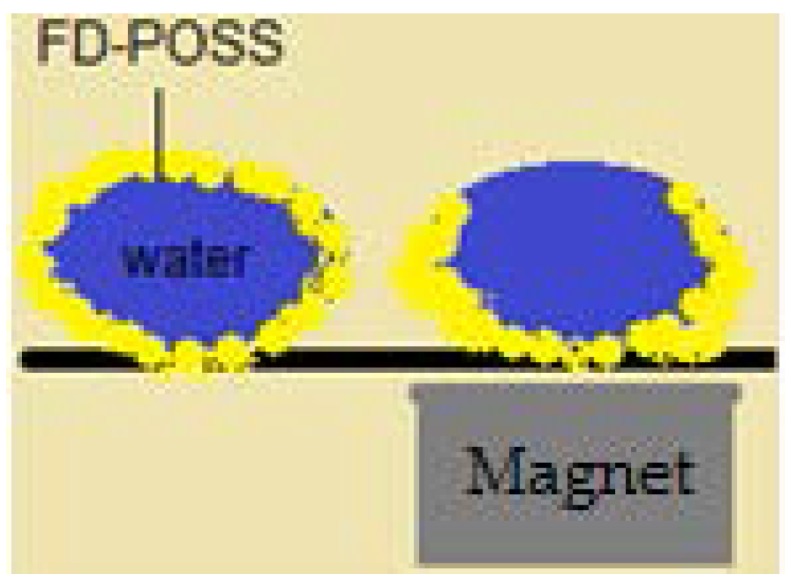
Liquid marble covered in FD-POSS. Migration of the powder towards the bottom, due to a magnetic field.

“Intelligent” liquid marbles, manipulated with the help of a magnet were obtained. By placing a magnet under the liquid marble, the powder from the top is attracted to the bottom of the drop, leaving an opening in the shell (Figure 20). 

**Figure 21:**
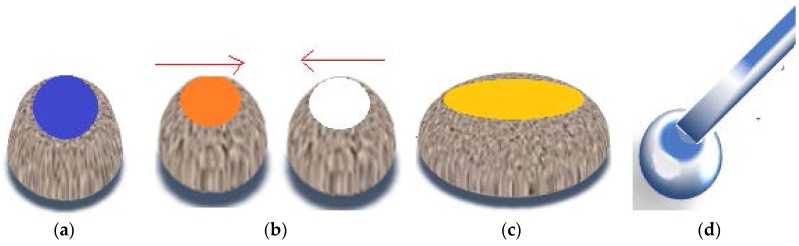
(**a**) Liquid marble open at the upper pole; (**b**,**c**) chemiluminescent reaction as a result of coalescence and new product formation; (**d**) chromatographic strip introduced into the internal phase (direct qualitative identification).

FD-POSS and FD-POSS /Fe_3_O_4_ hydrophobic nanoparticles are used to cover water drops, as displayed by Xue et al. in Figure 21a. Internal phases include: dimethylsulfoxide (DMSO), hexadecane, toluene, ethanol. These liquid marbles can be moved either direction, can be merged in order to induce coalescence, allow opening and subsequent closing (Figure 21b). This process allows coverage with another hydrophobic powder and reactant inoculation to obtain new products. Color shifts and chemiluminescence reactions are indicators of internal liquid marble activity (Figure 21). If a liquid marble is open at the top, a reaction product can be collected through capillarity, analyzed and identified through chromatographic methods. It can also be purified directly into the internal phase of the marble, using an alumina chromatographic strip, as presented by the same authors [25] in Figure 21d. 

Wang et al. [91] correlated the use of liquid marbles as biological reaction media with their application as chemical reactors. The experiments use magnetic lanthanide nanoparticle covered liquid marbles (UCNP-lanthanide-doped up conversion nanoparticles) capable of converting NIR light into visible (Vis) light. These nanoparticles transform low energy photons (NIR) into high energy photons (UV-Vis), and are usually transition metals, lanthanide or actinide ions, attached to an organic crystalline structure. The hexagonal stand of yttrium sodic fluorine (NaYF_4_) is a host to produce green light when enriched with Yb^3+^/Er^3+^; it also produces blue light when enriched with Yb^3+^/Tm^3+^. POSS is used for hydrophobization of UCNP. Liquid marbles formed by rolling in a bed of hydrophobic powder were exposed to NIR radiations. As a result, they generated visible green light and induced photocatalysis in the internal phase. When applying this treatment to protoporphyrin IX, oxygen reactive species (ROS) are formed. This would be impossible in the case of liquid marbles covered in ordinary shells, which don’t allow light passage. In the same study, first investigations were made with respect to cancer cells photodynamic therapy. Cancer cells were incubated in UCNP covered liquid marbles, along with protoporphyrin IX. It was proven that after irradiation, the cells manifested low viability, but only the ones incubated with protoporphyrin IX. It was made clear that UCNP-POSS covered liquid marbles have an important role in monitoring anti-cancerous therapies/drugs mechanisms.

### 5.5. Liquid Marbles as Sensors and Analytical Platforms

As previously presented, liquid marbles are considered microreactors, in terms of phenomena happening inside the liquid phase, after an external substance is added. Other internal changes are triggered by the permeability of the shell, transforming liquid marbles in sensors. This hypothesis comes as a result of chemical reactions or color modifications occurring into the internal phase. It is stated that liquid marbles act like stimuli-responsive formations [14]. The proof stands in PVDF covered liquid marbles, with an internal phase of ammonium acetate, acetic acid and acetylacetone solution, placed in a formaldehyde atmosphere. The color changes from white to yellow [92]. These liquid marbles function as sensors, by detecting gases. Silica covered liquid marbles with a copper chloride solution core, placed in an ammonia atmosphere, change color from blue-green to dark blue. Color intensity and rapid change are parameters to quantitative determine ammonia concentration [93,94]. “Plasmonic liquid marbles” covered in Ag/Au nanoparticles enriched with Raman spectroscopy, are precursors in obtaining special analytical platforms. They are used as qualitative detectors, but also as quantitative ones due to the possibility to trace chemical compounds, even in femto- or ato-molar concentrations (10^−15^, 10^−18^). This method is one of the most popular in detecting numerous analytes from waste samples recovered after industrial spills [95]. By increasing the liquid marbles’ internal phase volume, they undergo a lateral expansion. These formations no longer maintain a spherical shape, but rather acquire a “puddle” shape, characterized by a larger surface area to volume ratio. Thus, when referring to the corresponding “plasmonic liquid puddles”, advantages arise when using them as analytical platforms, due to their larger surface areas, which enable identifications and quantitative measurements [14]. The solution’s pH is another stimulus that liquid marbles are responsive to. Latex particles stabilized with polystyrene, distribute as surface chains when covering a solution with pH < 4. The shell maintains the drop’s integrity, at the surface of a pH > 4 solution. If the pH > 4 is alkalinized, liquid marbles collapse [96]. In environmentally friendly industries, stimuli responsive liquid marbles are used as cleaning agents. They maintain a spherical form until making contact with the pollutant agent. As a result, the shell breaks and a detoxifying solution acts. “Cleaning” liquid marbles include 1N-oxone as internal phase and Cab-O-Sil T-530 shells and are used to clean oily spill contaminated water, biological agents polluted air and to decontaminate equipment/ personnel from specialized institutions or as household pool cleaners [97].

### 5.6. Liquid Marbles as Gas Sensors, Transporters and Emitters: Gas Marbles

Through their porous shell, liquid marbles proved to be permeable to gases. This aspect was demonstrated through experiments carried out to reveal color changes of the marble’s core or collapse of the entire structure, as previously presented in Section 4.4. Color changes of indicator liquid marbles (CuCl_2_, CoCl_2_) in the presence of flexographic red ink liquid marbles confirm gas transportation and sensing. Besides qualitatively exploiting shell gas sensing properties, studies by Tian et al. were developed in order to prove the possibility of quantitative gas sensing using liquid marbles. An ammonia solution was used as internal phase. Gas emissions were measured from outside the liquid marbles using two colorimetric detection techniques. The first method implied UV-Vis spectrometric measurements of absorbance depending on concentration of CuCl_2_ indicator solution, after exposure to ammonia liquid marbles in a sealed environment. Color change was also observed. The second ammonia detection method used a paper-based gas sensing method, which proved to be preferred since it does not rely on Beer’s Law. This quantitatively gas sensing design is proposed as a low-cost vapor sensor with possible applications as water and soil gas emission evaluator. It can also function as an indicator of analytes of interest on certain surfaces [98].

Recent research reveals the discovery of marbles which are not liquid, but gaseous. The so called “gas marbles” are similar to liquid marbles, but much more resistant to external forces. Their outer shell consists of plastic microspheres packed together onto a liquid meniscus and are maintained so by surface tension. Resistance to needle piercing, inflation/deflation was proven under pressure 10 times bigger/lower than atmospheric normal conditions. Moreover, no bursting/collapse was observed during the experiments, until extreme conditions were reached. Further experiments will concern gas marbles’ external layer permeability for gasses with potential use in gas storage [99]. 

## 6. Conclusions

This review summarizes the latest and already known literature data regarding liquid marbles’ properties and preparation techniques. As they have proven versatility in formulation and also in means of use, these solid-fluid structures can ease and innovate many industrial/laboratory processes. Preliminary research was developed in order to establish laboratory conditions necessary to obtain liquid marbles, using as raw materials model active ingredients. Once the structures were formed, literature information regarding their properties was confirmed once more. 

Liquid marbles’ stability investigations and applications in the pharmaceutical area are topics proposed for future exploration, as they may be included in controlled release pharmaceutical forms. They are proposed as hollow granules and microcapsules precursors and as integrating parts of Pickering emulsions. A future concern regards stability assessment of liquid marbles and pharmaceutical forms in which they are intended to be included, in correlation with the formulation and the pursued therapeutic/cosmetic effect.
